# Neurocognitive Profiles of Mood Phases in Bipolar Disorder: Is Agitated Depression Related to Mania or Depression?

**DOI:** 10.1192/j.eurpsy.2025.436

**Published:** 2025-08-26

**Authors:** F. Bardi, A. Restaino, E. De Chiara, C. Calderoni, F. Grisoni, G. Mandracchia, A. M. D’Onofrio, S. Margoni, G. Sani, A. Simonetti

**Affiliations:** 1 Fondazione Policlinico Universitario Agostino Gemelli IRCCS; 2 Università Cattolica del Sacro Cuore, Rome, Italy

## Abstract

**Introduction:**

Agitated Depression (AgD) is a unique subtype of depression marked by impulsivity, higher suicide risk, treatment resistance, and worse clinical outcomes compared to Non-Agitated Depression (Non-AgD). Despite these clinical distinctions, the underlying neuropsychological mechanisms that differentiate AgD from Non-AgD remain poorly defined.

**Objectives:**

This study aims to explore the neurocognitive correlates that differentiate AgD from Non-AgD.

**Methods:**

The study cohort included 722 participants, divided into five groups: AgD, Non-AgD, subjects in a manic state (Mnc), euthymic subjects with bipolar disorder (Eu), and healthy controls (HC). All participants underwent a comprehensive neurocognitive assessment including the Wisconsin Card Sorting Test (WCST), the Interference Component of the Stroop Test (ST), the Semantic Fluency Test (SFT), the Trail-Making Test A and B (TMT-A, TMT-B), and Raven’s Progressive Matrices (RPM). Data were analyzed using one-way ANOVAs and Tukey post-hoc tests to compare cognitive performance across groups.

**Results:**

Non-AgD showed inferior performance compared to AgD on the WCST (non-perseverative errors: p=0.037; perseverative errors: p=0.010; categories identified: p=0.026), ST (*p*=0.000), TMT-A (*p*=0.046), and TMT-B (*p*=0.001). Non-AgD also underperformed Mnc at ST (*p*=0.002), SFT (*p*=0.025), TMT-A (*p*=0.007), TMT-B (*p*=0.005), and RPM (*p*=0.012). HC consistently outperformed AgD, Non-AgD, Mnc, and Eu individuals on all neurocognitive tests except for the WCST, where no significant differences were observed between HC, Eu, and AgD. Eu demonstrated superior performance on the WCST (*p*≤0.001), ST (*p*=0.000), and TMT (*p*=0.000) compared to Non-AgD, with no significant differences compared to AgD.

**Conclusions:**

The findings reveal distinct neurocognitive profiles for AgD and Non-AgD. The excitatory mechanisms associated with AgD may contribute to enhance attentional resources and cognitive flexibility but also greater impulse control difficulties. The neuropsychological profile of Eu patients resembles that of AgD, suggesting residual cognitive differences compared to HC. This study enhances our understanding of AgD by highlighting the differences in cognitive profiles of AgD, Non-AgD, Mnc, and Eu, and emphasizing the need of considering neurocognitive factors in the characterization and treatment of AgD.

**Disclosure of Interest:**

None Declared

